# SARS-CoV-2 complete genome sequencing from the Italian Campania region using a highly automated next generation sequencing system

**DOI:** 10.1186/s12967-021-02912-4

**Published:** 2021-06-05

**Authors:** Anna Maria Rachiglio, Luca De Sabato, Cristin Roma, Michele Cennamo, Mariano Fiorenza, Daniela Terracciano, Raffaella Pasquale, Francesca Bergantino, Ernesta Cavalcanti, Gerardo Botti, Gabriele Vaccari, Giuseppe Portella, Nicola Normanno

**Affiliations:** 1 Cell Biology and Biotherapy Unit, Istituto Nazionale Tumori “Fondazione Giovanni Pascale”, IRCCS, Via Mariano Semmola, 80131 Napoli, Italy; 2 ISS-Department of Food Safety, Nutrition and Veterinary Public Health, Istituto Superiore di Sanità, 00161 Rome, Italy; 3 Dipartimento Scienze Mediche Traslazionali, Università di Napoli “Federico II”, 80131 Napoli, Italy; 4 Laboratory Medicine Unit, Istituto Nazionale Tumori-IRCCS “Fondazione G. Pascale”, 80131 Napoli, Italy; 5 Scientific Direction, Istituto Nazionale Tumori-Irccs-Fondazione G. Pascale, 80131 Napoli, Italy

**Keywords:** Covid-19, SARS-CoV-2 genome, Next generation sequencing, Campania region

## Abstract

**Background:**

Since the first complete genome sequencing of SARS-CoV-2 in December 2019, more than 550,000 genomes have been submitted into the GISAID database. Sequencing of the SARS-CoV-2 genome might allow identification of variants with increased contagiousness, different clinical patterns and/or different response to vaccines. A highly automated next generation sequencing (NGS)-based method might facilitate an active genomic surveillance of the virus.

**Methods:**

RNA was extracted from 27 nasopharyngeal swabs obtained from citizens of the Italian Campania region in March–April 2020 who tested positive for SARS-CoV-2. Following viral RNA quantification, sequencing was performed using the Ion AmpliSeq SARS-CoV-2 Research Panel on the Genexus Integrated Sequencer, an automated technology for library preparation and sequencing. The SARS-CoV-2 complete genomes were built using the pipeline SARS-CoV-2 RECoVERY (REconstruction of COronaVirus gEnomes & Rapid analYsis) and analysed by IQ-TREE software.

**Results:**

The complete genome (100%) of SARS-CoV-2 was successfully obtained for 21/27 samples. In particular, the complete genome was fully sequenced for all 15 samples with high viral titer (> 200 copies/µl), for the two samples with a viral genome copy number < 200 but greater than 20, and for 4/10 samples with a viral load < 20 viral copies. The complete genome sequences classified into the B.1 and B.1.1 SARS-CoV-2 lineages. In comparison to the reference strain Wuhan-Hu-1, 48 total nucleotide variants were observed with 26 non-synonymous substitutions, 18 synonymous and 4 reported in untranslated regions (UTRs). Ten of the 26 non-synonymous variants were observed in ORF1ab, 7 in S, 1 in ORF3a, 2 in M and 6 in N genes.

**Conclusions:**

The Genexus system resulted successful for SARS-CoV-2 complete genome sequencing, also in cases with low viral copies. The use of this highly automated system might facilitate the standardization of SARS-CoV-2 sequencing protocols and make faster the identification of novel variants during the pandemic.

**Supplementary Information:**

The online version contains supplementary material available at 10.1186/s12967-021-02912-4.

## Background

Coronavirus disease-19 (COVID-19), declared as pandemic on March 2020 by WHO, is an infectious disease caused by the Severe Acute Respiratory Syndrome coronavirus 2 (SARS-CoV-2). COVID-19 represents a global public health concern due to the relatively large fraction of infected people who develop a severe and often fatal interstitial pneumonia [1–3]. Indeed, as of December 2019, SARS-CoV-2 infected more than 114 million individuals worldwide, causing more than 2.5 million deaths [4].

Italy was the first European country to be hardly hit by the SARS-CoV-2 epidemic. The first wave of infection mainly affected the Northern Italian regions, causing thousands of deaths, especially among the most fragile individuals [5]. As of March 1, 2021, Italy has been affected by 2,955,434 cases and 98,288 deaths (www.salute.gov.it).

Phylogenetically, SARS-CoV and SARS-CoV-2 share a most recent common ancestor and are classified within the subgenus *Sarbecovirus* in the genus *Betacoronavirus* [6]. The SARS-CoV-2 genome size varies from 29.8 to 29.9 kb. Its genomic organization consists of 10 open reading frames (ORFs) coding for ORF1ab polyproteins, spike (S), envelope (E), membrane (M), nucleocapsid (N) and accessory proteins [7].

Since the first description of the SARS-CoV-2 sequences in December 2019, an exponentially increasing number of sequences of the virus have been reported with over 550,000 complete genomes deposited in the GISAID database [8]. Based on mutation position and phylogenetic analysis, the SARS-CoV-2 complete genome has been classified in two major lineages, named A and B, and several sublineages [9]. The D614G mutation affecting the spike glycoprotein of SARS-CoV-2 strains, characterizes the B.1 lineage, which originated from southern Europe and become rapidly the most prevalent lineage worldwide. This variant has an increased infectiveness and allows fast spreading of the virus [10, 11]. The number of SARS-CoV-2 variants reported has increased exponentially. The variant B.1.1.7, recently identified in United Kingdom (UK), is rapidly becoming the most prevalent variant in many European countries [9]. In South Africa, the B.1.351 emerged independently from B.1.1.7 [12], while the P.1 variant has been isolated in Brazil [13].

The possibility that new variants will emerge in the next future with greater transmissibility, a different clinical pattern and a possible difference in the response to vaccines and antibody-based therapies, makes an active genomic surveillance of the virus essential. The development of a genomic surveillance practice requires the availability of standardized sequencing protocols, which can be easily implemented in virology diagnostic laboratories and therefore capable of generating robust data that can be immediately transferred for planning health interventions. In this respect, next generation sequencing (NGS) can provide high-quality, full-scale sequences of viral isolates collected from infected individuals. Analyses of whole genome sequences can provide insight into the pattern of distribution of specific variants and the dynamics of genetic diversity during epidemics.

In this study, we present the results of an evaluation of the highly automated sequencer Genexus for the determination of the SARS-CoV-2 genomics sequence. For this validation, we used samples from the first wave of the pandemic in the Campania region in Italy, thus providing for the first time data on the progress of the epidemic in a region with low levels of spread of the virus in the first half of 2020.

## Methods

### Patients and samples

The SARS-CoV-2 RNA samples were collected at Department of Translational Medical Sciences—University of Napoli “Federico II”. The samples were obtained from nasopharyngeal swabs of 27 individuals who tested positive by standard SARS-CoV-2 diagnostic Real-Time PCR test during the period between 12 March and 27 April 2020 (Table 1).

**Table 1:** Demographic and epidemiologic characteristics

Sample ID	Collection date	Municipality	Patient condition	Contacts	Age	Sex
p01	02/04/2020	Napoli	Intensive therapy	–	72	M
p03	01/04/2020	Napoli	Mild symptoms	p34	47	M
p04	31/03/2020	Volla	Asymptomatic	–	54	M
p06	25/03/2020	Napoli	Asymptomatic	–	48	F
p07	03/04/2020	Napoli	Intensive therapy	–	66	M
p09	03/04/2020	Napoli	Mild symptoms	–	56	M
p11	30/03/2020	Napoli	Asymptomatic	–	27	F
p12	03/04/2020	Napoli	Asymptomatic	–	62	M
p14	30/03/2020	Napoli	Mild symptoms	–	59	F
p15	30/03/2020	Napoli	Asymptomatic	–	57	M
p17	22/04/2020	Napoli	Sub intensive therapy	–	75	M
p19	22/04/2020	Napoli	Sub intensive therapy	–	84	F
p24	22/04/2020	Napoli	Sub intensive therapy	–	71	M
p31	30/03/2020	Casavatore	Asymptomatic	–	60	F
p34	12/03/2020	Napoli	Mild symptoms	p3	61	M
p36	22/03/2020	Aversa (CE)	Mild symptoms	–	58	M
p37	19/03/2020	Gricignano (CE)	Mild symptoms	–	23	M
p38	03/04/2020	Napoli	Intensive therapy	–	78	M
p39	22/03/2020	Casaluce (CE)	Mild symptoms	–	26	M
p40	31/03/2020	Napoli	Asymptomatic	–	51	F
p41	04/03/2020	Napoli	Intensive therapy	–	67	F
p42	19/03/2020	Marcianise (CE)	Mild symptoms	–	83	F
p44	30/03/2020	Boscotrecase	Asymptomatic	–	35	F
p45	03/04/2020	Napoli	Asymptomatic	–	42	F
p59	30/03/2020	Napoli	Mild symptoms	–	33	M
p60	22/03/2020	Napoli	Sub intensive therapy	–	67	F
p61	30/03/2020	Napoli	Mild symptoms	–	61	M

The patients were all from the city of Napoli (NA) and nearby towns, with the exception of four individuals from small towns in the province of Caserta (CE). Eleven were females and 16 males, with a median age of 56 years (range 23–84). Nine cases were asymptomatic at the time of the swab, 10 with mild symptoms and 8 in sub-intensive or intensive therapy units. Two cases (p03 and p34) both with mild symptoms were tested following a close contact.

RNA samples were extracted from 1 ml of Nasopharyngeal swab using the CE marked Abbott Sample Preparation System (Abbott Laboratories, Abbott Park, IL), an iron particle-based method for RNA preparation. To detect viral RNA, the samples were analysed using Abbott Real Time SARS-CoV-2 assay, a real-time reverse transcription polymerase chain reaction (rRT-PCR) test on the Abbott m2000 System, following the manufactures instructions. The Abbott Real Time SARS-CoV-2 assay is a dual target assay for the RdRp and N genes. The two SARS-CoV-2-specific probes are labelled with the same fluorophore. The assay has been reported to detect 100 viral copies/ml (3.1 genome equivalent/reaction) with 95.2% sensitivity.

The samples used for this technology assessment were fully anonymized. The research protocol was approved by the Institutional Review Board (IRB) of the Istituto Nazionale Tumori “Fondazione Giovanni Pascale”.

### Quantification of SARS-CoV-2

The extracted RNAs were quantified using the Qubit RNA High Sensitivity Kit (Invitrogen, USA). The number of viral copies were assessed with the TaqPath™ COVID-19 RT-PCR Kit (ThermoFisher Scientific, San Diego, CA), according to the manufacturer’s protocol.

For quantification of SARS-CoV-2 copies by real-time PCR, a calibration curve was obtained from serial tenfold dilutions of the standard TaqPath™ COVID-19 Control. The assay was performed on AB ViiA 7 Dx Real-Time PCR System (ThermoFisher Scientific, San Diego, CA). Ct values for samples and controls were determined from amplification curves calculated from the ΔRn value of the 6-carboxyfluorescein (FAM)/6-carboxy-X-rhodamine (ROX) signals, using the ViiA 7 software. The automatic analysis function of the ViiA 7 software was used to set the background and threshold values.

### Next generation sequencing of SARS-CoV-2

For the whole viral genome sequencing, a volume of 25 µl of a tenfold dilution of each sample was distributed in an Applied Biosystems™ MicroAmp™ EnduraPlate™ Optical 96-Well Clear Reaction Plate. The plate was sealed with a sheet of adhesive PCR plate foil and was placed on the Ion Torrent Genexus Integrated Sequencer (ThermoFisher Scientific, San Diego, CA).

All the following steps, including cDNA synthesis, library preparation and equalization, template preparation, sequencing and post-run analysis were fully automated on the Genexus system.

The samples were sequenced on two Ion Torrent™ GX5™ Chip. The sequencing run was performed by using the Ion AmpliSeq SARS-CoV-2 Research Panel on the Genexus Integrated Sequencer (ThermoFisher Scientific, San Diego, CA), according to two preinstalled assays: “SARS-CoV-2 Low Titer Research Assay” for samples with viral load < 200 copies and “SARS-CoV-2 Research Assay” for > 200 copies.

The Ion AmpliSeq SARS-CoV-2 Research Panel consists of 2 primer pools targeting 237 amplicons tiled across the SARS-CoV-2 genome, with an additional 5 primer pairs targeting human expression controls. The SARS-CoV-2 amplicons range from 125 to 275 bp in length.

Post-sequencing run analysis was performed by Genexus Software with following plug-in: COVID19AnnotateSnpEff for variant annotation, IRMA for genome-assisted and Trinity for de novo sequence assembly.

### Complete genome reconstruction

Complete genome sequences were quality checked using the FastQC tool and built using the pipeline SARS-CoV-2 RECoVERY 3.0 (REconstruction of COronaVirus gEnomes & Rapid analYsis) implemented in the Galaxy platform for computational research [14].

### Phylogenetic analysis

The Maximum Likelihood tree was built using 133 SARS-CoV-2 Italian complete genomes collected during the same period of the samples from this study and downloaded from the GISAID database [8] with the 21 Italian complete genomes here reported. The phylogenetic tree was built using IQ-TREE (v.1.6.10) [15] with the best fit model indicated by the Model Finder implemented in IQ-TREE and 1000 bootstrap replicates. The Mutation Browser v-1.3 database was used to compare the variants found in the complete genomes from GISAID database with those reported in our study [16].

### S5 XL sequencing analysis

In order to confirm the unreported variants detected by Genexus, we performed an independent sequencing analysis using Ion S5 XL system.

The cDNA was prepared using 7 µl of viral RNA by Invitrogen™ SuperScript™ VILO™ cDNA Synthesis Kit (Thermo Fisher Scientific, San Diego, CA).

Libraries were prepared using the Ion AmpliSeq SARS-CoV-2 Research Panel according to the Ion AmpliSeq™ Library Kit 2.0 user guide (Thermo Fisher Scientific, MAN0006735 rev F.0) and all the reactions were performed in an Applied Biosystems™ Veriti™ 96-Well Thermal Cycler (Thermo Fisher Scientific, San Diego, CA). The final concentration of each cDNA library was determined on the Agilent 2100 system by the Agilent High Sensitivity DNA Assay (Agilent Technologies, Santa Clara, CA), following the manufacturer recommendations. Barcoded libraries were diluted to 30 pM, pooled in equal volume aliquots, and then loaded on to the Ion Chef™ Instrument (Thermo Fisher Scientific, San Diego, CA) for emulsion PCR, enrichment, and loading onto the Ion S5 520 chip. Two sequencing runs were performed on the Ion S5 XL System (Thermo Fisher Scientific, San Diego, CA). Reads were aligned with the Wuhan-Hu-1 NCBI Reference Genome (Accession number: MN908947.3) on the Torrent Suite v. 5.12.1.

### Sanger sequencing

In addition, as proof of concepts, for 3 samples in which are available viral RNA we confirmed the variants also by Sanger method. The mutational sites analysed were: position 2488 and 2498 within orf1ab, 22,629 within spike and 25,621 within orf3a gene.

The genomic region harbouring mutational sites was amplified using the following primers: orf1ab_Fw (5′-*TGTAAAACGACGGCCAGT*AAGCCTTGAATTTAGGTGAAACA-3′) and orf1ab_Rw (5′-*CAGGAAACAGCTATGACC*ATTAGGTGCAAGGGCACAGT-3′); spike_Fw (5′-*TGTAAAACGACGGCCAGT*AACTTTAGAGTCCAACCAACAGAA-3′) and spike_Rw (5′-*CAGGAAACAGCTATGACC*CCTGGAGCGATTTGTCTGA-3′); orf3_Fw (5′-*TGTAAAACGACGGCCAGT*CGTTGCACTTCTTGCTGTTT-3′) and orf3_Rw (5′-*CAGGAAACAGCTATGACC*GCAAAGCCAAAGCCTCATTA-3′) to obtain respectively 317 bp, 290 bp and 263 bp amplicons. The nucleotides presented in italic type correspond to M13 consensus sequences and were used also for cycle sequencing with M13 consensus primers. PCR was performed in 50 µl reaction volume containing 1× AmpliTaq® Gold DNA polymerase buffer (Thermo Fisher Scientific, San Diego, CA); 2.5 mM MgCl_2_; 0.02 mM each deoxynucleotide; 0.2 µM each primer; 2.5 units AmpliTaq® Gold DNA Polymerase (Thermo Fisher Scientific, San Diego, CA) and 2 µl of cDNA template. The cDNA was prepared using 7 µl of viral RNA by Invitrogen™ SuperScript™ VILO™ cDNA Synthesis Kit (Thermo Fisher Scientific, San Diego, CA). PCR reactions were performed by incubating the samples at 95 °C for 10 min, followed by 40 cycles of 95 °C for 30 s, 58 °C for 30 s and 72 °C for 1 min. The final extension step was performed for 10 min at 72 °C and the samples were then chilled to 4˚C. PCR reactions were run in a Applied Biosystems™ Veriti™ 96-Well Thermal Cycler (Thermo Fisher Scientific, San Diego, CA). The PCR products were electrophoresed in an agarose gel to confirm successful amplification.

Following PCR, amplification products were purified using the pre-sequencing Kit Illustra™ ExoProStar™ (GE Healthcare, Chicago, IL). Sequencing reactions were performed with the BigDye® Terminator Cycle Sequencing Kit v1.1 (Thermo Fisher Scientific, San Diego, CA) chemistry using both M13-F and M13-R sequencing primers to obtain forward and reverse sequences. Cycle Sequencing reactions were cleaned up using BigDye Terminator purification kit (Thermo Fisher Scientific, San Diego, CA). Purified sequencing reactions were analyzed using the Applied Biosystems 3500 Genetic Analyzer. The sequence data were analysed using the Sequencer software Ver. 4.10.1 (Gene Codes Corporation, Ann Arbor, USA).

## Results

### SARS-CoV-2 RNA quantification

Quantification of RNA extracted from swabs with the Qubit RNA High Sensitivity Kit (Invitrogen, USA) was possible only for 8/27 samples, due to the too low RNA concentrations of most samples (Table 2).

**Table 2:** Results of SARS-CoV-2 sequencing by Ion Torrent Genexus Integrated system

Sample ID	RNA quantification		No. viral copies sequenced (25 μl)	Total reads	Mapped reads (%)	% complete genome	Sequence name
	Qubit ng/μl	Real time PCR copies/μl					
p01	Too low	< 20	–	104,239	69,815 (66.9)	100	CAM-INTPas-14
p03	Too low	745	1863	1,710,451	1,645,209 (96.2)	100	CAM-INTPas-1
p04	Too low	60	150	431,728	323,912 (75.0)	100	CAM-INTPas-16
p06	1.28	51,184	127,960	2,467,092	2,323,266 (94.2)	100	CAM-INTPas-2
p07	Too low	462	1,155	1,190,112	1,127,406 (94.7)	100	CAM-INTPas-3
p09	Too low	< 20	–	60,739	6391 (10.5)	84	^a^
p11	Too low	1049	2623	1,207,327	1,133,720 (93.9)	100	CAM-INTPas-4
p12	Too low	< 20	–	181,669	156,664 (86.2)	100	CAM-INTPas-15
p14	1.51	< 20	–	79,427	2003 (2.5)	63.0	^a^
p15	Too low	< 20	–	317,588	7843 (2.4)	95.0	^a^
p17	Too low	1223	3058	135,720	124,336 (91.6)	100	CAM-INTPas-21
p19	0.84	4180	10,405	748,062	724,073 (96.8)	100	CAM-INTPas-5
p24	Too low	848	2120	1,535,836	1,467,096 (95.5)	100	CAM-INTPas-6
p31	1.1	319	798	433,020	381,523 (88.1)	100	CAM-INTPas-7
p34	1.31	5435	13,588	1,502,575	1,461,532 (97.2)	100	CAM-INTPas-8
p36	1.19	< 20	–	99,135	24,489 (24.7)	95.0	^a^
p37	Too low	203,140	507,850	1,776,733	1,624,345 (91.4)	100	CAM-INTPas-9
p38	Too low	79,570	198,925	300,518	261,815 (87.1)	100	CAM-INTPas-10
p39	1.01	15,806	39,515	2,242,528	2,082,937 (92.8)	100	CAM-INTPas-11
p40	Too low	24	59	188,148	129,527 (68.8)	100	CAM-INTPas-17
p41	Too low	3435	8588	1,852,539	1,759,592 (94.9)	100	CAM-INTPas-12
p42	Too low	< 20	–	781,315	656,572 (84.0)	100	CAM-INTPas-18
p44	Too low	< 20	–	597,949	367,619 (61.5)	100	CAM-INTPas-19
p45	Too low	360	900	257,329	223,036 (86.6)	100	CAM-INTPas-20
p59	Too low	911	2278	1,428,577	1,383,059 (96.8)	100	CAM-INTPas-13
p60	5.52	< 20	–	1,202,245	7406 (0.6)	94.0	^a^
p61	Too low	< 20	–	96,345	8783 (9.11)	81.0	^a^

^a^Not submitted into the GISAID database

The analysis performed with the TaqPath™ COVID-19 RT-PCR test found that nine samples had a viral load > 1000 copies/µl (range 1049–203,140), six had a load between 200 and 1000 copies/µl (range 319–911) and two samples carried < 200 copies/µl of the SARS-CoV-2 viral genome (values 24 and 60). Ten samples had viral loads < 20 copies (Table 2).

### Sequencing of SARS-CoV-2 with the Ion Torrent Genexus Integrated system

We used the SARS-CoV-2 Research Assay protocol for sequencing the 15 samples with a high viral titer (> 200 copies/µl) while the SARS-CoV-2 Low Titer Research Assay protocol was employed for the remaining samples with low (< 200 copies/µl) viral titer.

The Ion Sphere Particles (ISP) loading was 92.83% for the SARS-CoV-2 Research Assay run and 90.1% for the SARS-CoV-2 Low Titer Research Assay run. In addition, 99.89% of the ISP was represented by libraries for both assay protocols. These results show the good quality of the sequenced libraries in both standard and low titer protocols, thus demonstrating the high performance of the panel in terms of target amplification and sequencing specificity.

All samples returned reads mapping to the 5 human expression controls of the Ion AmpliSeq SARS-CoV-2 Research Panel, indicating that automated library generation on the Genexus Integrated Sequencer was successful.

Reads not mapped to the human expression controls were mapped against the SARS-CoV-2 reference sequence to determine percent base reads on target. The mean percent of reads on target for the 12 cases with low viral titer was 41.53% (range 5–98%) with a median value of 36% [standard deviation (s.d.) ± 41] (Table 2). In contrast, the mean value for samples with high titer was 95.6% (range 80.6–99.8%) with a median of 98.2% (s.d. ± 8).

The complete genome (100%) of SARS-CoV-2 was successfully obtained for 21 samples with a mean coverage > 100× (max coverage 13,393×; min coverage 142×) (s.d. ± 4.378). In particular, the complete genome was fully sequenced for all samples with high viral titer (> 200 copies), for the two samples with a viral genome copy number < 200 but greater than 20, and for 4/10 samples with viral load < 20 (Table 2).

### Phylogenetic analysis

Phylogenetic analysis was performed on 21 sequences (Table 3). In comparison to the reference strain Wuhan-Hu-1 (Accession number: NC_045512.2), 48 total variants were observed with 26 non-synonymous substitutions, 18 synonymous and 4 reported in untranslated regions (UTRs) (Table 3). Ten of the 26 non-synonymous were observed in ORF1ab, 7 in S, 1 in ORF3a, 2 in M and 6 in N genes.

**Table 3:** Variants of Italian SARS-CoV-2 complete genome

Gene name	Position	Mutation	Codon change	Amino acids change	No. Italian samples	Reported before
5′ UTR	241		C/T		21	Yes
orf1ab nsp1	586	Synonymous	ctT/ctC	L107	1	Yes
orf1ab nsp2	1117	Synonymous	atT/atC	I104	1	Yes
orf1ab nsp2	1191	Nonsynonymous	cCa/cTa	P129L	2	Yes
orf1ab nsp2	1524	Nonsynonymous	tGt/tAt	C240Y	1	Yes
orf1ab_nsp2	2488	Synonymous	ttC/ttT	F561	1	No
orf1ab nsp2	2498	Nonsynonymous	Gaa/Aaa	E565K	3	No
orf1ab nsp3	3037	Synonymous	ttC/ttT	F106	21	Yes
orf1ab nsp3	4235	Nonsynonymous	Aat/Gat	N506D	1	Yes
orf1ab nsp3	4255	Synonymous	ccG/ccT	P512	3	Yes
orf1ab nsp3	5812	Synonymous	gaC/gaT	D1031	1	Yes
orf1ab nsp3	6629	Nonsynonymous	Ctt/Ttt	L1304F	1	Yes
orf1ab nsp3	7094	Nonsynonymous	Act/Gct	T1459A	1	No
orf1ab nsp4	9157	Synonymous	ttT/ttC	F201	1	Yes
orf1ab nsp6	11,008	Synonymous	caC/caT	H12	1	No
orf1ab nsp6	11,083	Nonsynonymous	ttG/ttT	L37F	1	Yes
orf1ab nsp12	13,748	Nonsynonymous	Agt/Ggt	S103G	2	Yes
orf1ab nsp12	14,408	Synonymous	Cta/Tta	L323	21	Yes
orf1ab nsp12	15,243	Nonsynonymous	tGt/tAt	C601Y	1	Yes
orf1ab nsp13	16,456	Nonsynonymous	Tca/Cca	S74P	1	Yes
orf1ab nsp15	19,839	Synonymous	aaT/aaC	N73	2	Yes
orf1ab nsp15	20,380	Synonymous	Cta/Tta	L254	1	No
S	21,566	Nonsynonymous	Ttt/Ctt	F2L	1	No
S	21,575	Nonsynonymous	Ctt/Ttt	L5F	2	Yes
S	22,629	Nonsynonymous	aAg/aGg	K356R	1	No
S	23,403	Nonsynonymous	gAt/gGt	D614G	21	Yes
S	23,422	Synonymous	gtC/gtT	V620	2	Yes
S	23,569	Synonymous	ggT/ggC	G669	1	No
S	23,575	Synonymous	tgC/tgT	C671	1	Yes
S	23,589	Nonsynonymous	aCt/aTt	T676I	1	No
S	23,675	Nonsynonymous	Gtt/Ttt	V705F	1	Yes
S	23,849	Synonymous	Tta/Cta	L763	2	Yes
S	25,135	Nonsynonymous	aaG/aaT	K1191N	1	Yes
ORF3a	25,621	Nonsynonymous	Gtt/Ttt	V77F	1	No
UTR ORF3a-M	26,522		C/T		2	No
M	26,530	Nonsynonymous	gAt/gGt	D3G	3	Yes
M	26,858	Synonymous	ttC/ttT	F112	1	Yes
M	26,915	Synonymous	agA/agG	R131	1	No
M	27,046	Nonsynonymous	aCg/aTg	T175M	1	Yes
N	28,514	Nonsynonymous	Gat/Tat	D81Y	1	Yes
N	28,881	Nonsynonymous	aGG/aAA	R203K	4	Yes
N	28,882	Synonymous	AgG/agA	R203	4	Yes
N	28,883	Nonsynonymous	Gga/Cga	G204R	4	Yes
N	28,902	Nonsynonymous	aTg/aCg	M210T	1	Yes
N	28,985	Nonsynonymous	Ggc/Tgc	G238C	1	Yes
N	29,009	Nonsynonymous	Gtc/Atc	V246I	1	Yes
3′ UTR	29,686		C/T		2	Yes
3′ UTR	29,823		T/A		1	Yes

The ORF1ab is the longest gene of CoV genome (21,289/29,903 bp) and is cleaved into non-structural proteins (NSP1-NSP16). Among NSPs, the NSP2 and NSP3 had the highest number of variants with 5 and 7 mutations, respectively.

The most common variants were the C241T and the C3037T in NSP3, the C14408T in NSP12 within the ORF1ab, and the D614G within the S protein. We compared the variants revealed in our complete genomes with those reported in the SARS- CoV-2 Mutation Browser v-1.3 database, containing sequence analysis of 10,416 SARS-CoV-2 strains from 111 locations and 6678 mutating positions. Interestingly, 12 variants were never detected before, of which 5 were reported in ORF1ab and 4 in S gene (Table 3). We could confirm 11/12 previously unreported variants by resequencing samples with available RNA by using an S5 XL apparatus (Additional file 1: Table S1). Sequencing of the p12 sample carrying the F2L variant in the S protein failed. In addition, the variants F561, E565K in orf1ab nsp2, K356R in the spike gene and V77F in orf3 were also confirmed by Sanger sequencing in samples with available RNA.

Following the GISAID classification, the variants C241T, C3037T, A23403G are the most common detected in several SARS-CoV-2 isolates throughout Europe. These mutations are characteristic of clade G and comprises the large Italian outbreak (since 29/01/2020 and still ongoing). Seventeen Italian complete genomes from this study showed the three mutations suggesting their classification in the clade G, lineage B.1. In addition, four sequences showed an additional mutation (G28882A) suggesting the classification in the clade GR, lineage B.1.1 originated from B.1, another clade mostly reported in Europe and in Italy. The classification within the G and GR clade was confirmed by phylogenetic analysis. The maximum likelihood tree showed 9 clusters within the G clade and 3 clusters within the GR clade.

Within the G clade, 13 sequences did not cluster with any other sequence. Three were reported from the municipality of Napoli and did not cluster with sequences from this study (p01, p12, p38).

One cluster showed sequences from the municipality of Napoli, p17 and p07, while p40 and p04 were from different municipalities. The major cluster with sequences from this study had 6 sequences represented by p11, p03, p41, p06, p59 from Napoli and the p37 sequence out of the sub-cluster from the province of Caserta. Two clusters showed the presence of sequences reported in other Italian regions. The p44 strain clustered with hCoV-19/Italy/LAZ-INMI-8/2020 (EPI_ISL_424342) from Central Italy (Lazio region), hCoV-19/Italy/VEN-UniVR-6/2020 (EPI_ISL_492985) and hCoV-19/Italy/LOM-UniMI-L160/2020 (EPI_ISL_542155) collected at the beginning of March in Northern Italy (Veneto and Lombardia regions).

The p19 and p24 showed evolutionary correlation with sequences from Lazio, Marche and Abruzzo regions collected in March 2020: hCoV-19/Italy/LAZ-INMI-9/2020 (EPI_ISL_424343), hCoV-19/Italy/MAR-UnivPM-78955-2/2020 (EPI_ISL_516088), hCoV-19/Italy/LAZ-INMI11-B/2020 (EPI_ISL_451304), hCoV-19/Italy/ABR-IZSGC-TE7097/2020 (EPI_ISL_528929) with p34 and hCoV-19/Italy/ABR-IZSGC-TE5472/2020 (EPI_ISL_420564) out of the former cluster.

Within the clade GR, one sequence did not cluster (p39) while p42 clustered with hCoV-19/Italy/LOM-UniMI-L182/2020 (EPI_ISL_542173) from Northern Italy (Lombardia region) and hCoV-19/Italy/SAR-AMVRC-28/2020 (EPI_ISL_458085) from Central Italy (Sardinia region). The p31 and p45 from different municipalities clustered together.

**Fig. 1:**
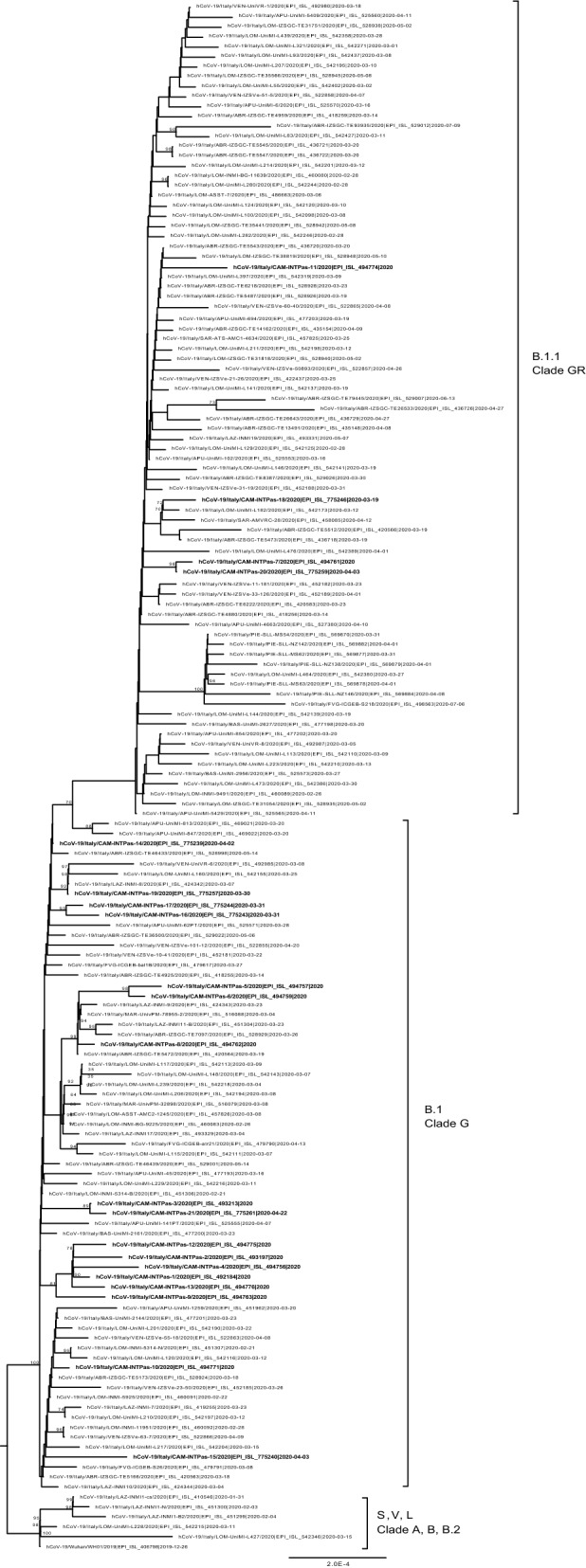
Maximum likelihood tree built using 133 SARS-CoV-2 complete genomes downloaded from GISAID database and 21 Italian strains reported in bold. The phylogenetic was built using IQ-TREE using the best fit model indicated by the Model Finder implemented in IQ-TREE and 1000 bootstrap replicates. Bootstrap > 70 are reported at nodes

The variants shared by p03 and p34, who have history contact, were inspected. The p34 sample shared two variants, P512 in ORF1ab NSP3 and D3G in M, with p19 and p24 samples, whereas p03 did not show these mutations, confirming the different origin of viral infection between the two cases. Based on the phylogenetic analysis related sequences were reported from different municipalities and the large municipality of Napoli showed the circulation of several viral sequences with point mutations shared within strains phylogenetically correlated or not. In addition, as reported in Table 1, p03 and p34 declared close contacts among them, however, they clustered in different position within the phylogenetic tree and showed different point mutations (Fig. 1).

## Discussion

Since the first complete genome sequencing of SARS-CoV-2 on 31 December 2019, and the first Italian case of COVID-19 in Italy [17], more than 550,000 complete genomes have been sequenced worldwide and released on GISAID database after 1 year of pandemic. To date, the most used and successful sequencing method to obtain complete genome is NGS. We present here the complete sequencing of SARS-CoV-2 genomes using the Ion Torrent Genexus System, a highly automated sequencer. In this study, 21 out of 27 SARS-CoV-2 RNA, tested with Genexus System, were fully sequenced.

The 6 samples not fully sequenced had a number of copies lower than the limit of quantification of the Real Time PCR assay (20 copies) and 3 of them had too low RNA for the Qubit quantification. The results suggest that samples had an amount of viral RNA closer to the limit of sequencing potential of the amplicon-based Ampliseq technology. However, despite the low number of copies and RNA, too low to be detected by the Qubit, four samples were fully sequenced suggesting that other factors related to the quality of the sample may also affect the success of library preparation and sequencing reaction.

The results obtained, however, highlight the potentiality of the Genexus System: (i) the users are involved only in the sample preparation and quantification, (ii) the automated process allows the users to focus on NGS raw data check and subsequent bioinformatic analysis, (iii) the technology allowed the complete genome sequencing of 78% of the samples (21/27) obtained from routine SARS-CoV-2 diagnosis process, with additional 3 samples showing nearly completed sequencing of the genome (> 95%). Moreover, the Genexus System permits the sequencing of 32 multiplexed samples in less than 24 h, representing a useful method for SARS-CoV-2 surveillance during a pandemic event. The Genexus System could be useful to analyse in a few days, respect to the Sanger sequencing, several viral strains from patients with an abnormal clinical presentation such as a very late viral clearance or to identify possible new variants in situations of rapid increase of contagions.

The complete genome sequences obtained were analysed by phylogenetic analysis and compared to the 133 Italian complete genomes on GISAID database collected in the same period of the samples analysed in this study. The sequences here reported were collected in March and April 2020 from nasopharyngeal swabs from Napoli province and nearby towns in the Campania region. The sequences clustered within the two lineages, B.1 and B.1.1, which were mostly detected in Italy and worldwide since February 2020, when the D614G mutation of the B.1 lineage was reported for the first time.

The Italian sequences within the B.1 formed 9 clusters while other 3 clusters were in B.1.1, confirming the heterogeneity of circulating strains. In addition, most of the sequences under study (14/21) were collected from Napoli and formed 7 different clusters consistent with an area with high population density. Two clusters (e.g.: p19, p44) were formed by strains from this study, showing evolutionary correlation to SARS-CoV-2 sequences reported in other Italian regions and suggesting common origin of viral strains. The cluster formed by p40 and p04 sequences also showed the circulation of correlated sequences in different municipalities suggesting intra-municipality commutes.

To date the only differences reported among SARS-CoV-2 are point mutations, excepting the deletion described in the B.1.1.7 and P.1, reported for the first time in December 2020 in the United Kingdom and Brazilian variants. The point mutations reported in this study and shared by all complete genomes are related to the B.1 and B.1.1 lineages as the D614G.

Other mutations were reported in one or two sequences only and never reported before. Since these mutations never fixed in the viral population, we can speculate that a correlation between these SNPs and viral adaptation may exist. The genes with the high number of point mutations were the spike with 11, 6 in nsp3 and 7 in N, three genes under positive selection [18, 19].

It is interesting to note that two patients who tested positive after a business meeting (p03 and p34) actually have genomic sequences not closely correlated, thus suggesting different origins of the infection. The sequencing of the viral genome can therefore also better clarify some dynamics relating to the spread of the infection in hospitals or in communities.

Since the appearance of SARS-CoV-2 several mutations have been reported in the spike gene and novel mutations are continuously described [10, 20–23]. Selected mutations such as the D614G might provide an advantage to the virus by increasing the cellular infectivity and virus transmissibility. Recently, a N501Y mutation on the Spike gene has been reported [24] showing a higher affinity to human ACE2 protein as compared to D614G. The surveillance on circulating strain is highly relevant today because the novel variants, with multiple mutations in their spike glycoproteins, are key targets of virus-neutralizing antibodies and raise the concern of vaccine efficacy against the novel strains.

## Conclusions

During a pandemic event the surveillance of circulating strains is crucial to understand the evolution of viral strains and the emerging of novel variants. This study is a first observation of variants detected in the Campania region; a region less affected than Italian Northern regions in the first phase of the pandemic in Italy. In particular, we reported the circulation of different variants within the Napoli province and the heterogeneity of different strains circulating between and within municipality. In addition, a novel automated technology as the Ion Torrent Genexus Integrated system allowed complete genome sequencing even of samples with relatively low viral titer in a relatively short timeframe, thus facilitating the continuous surveillance of novel variants.

## Supplementary Information

**Additional file 1: Table S1.** Confirmation of unreported variants by S5 and Sanger sequencing.

## Data Availability

The datasets used and/or analyzed during the current study are available from GISAID database (https://www.gisaid.org).

## Abbreviations

COVID-19: Coronavirus disease-19
SARS-CoV-2: Severe Acute Respiratory Syndrome coronavirus 2
NGS: Next Generation Sequencing
MERS-CoV: Middle East Respiratory Syndrome Coronavirus
ORFs: Open reading frames
S: Spike
E: Envelope
M: Membrane
N: Nucleocapsid
FAM: 6-Carboxyfluorescein
ROX: 6-Carboxy-X-rhodamine
LOD: Limit of detection
RECoVERY: REconstruction of COronaVirus gEnomes & Rapid analysis
UTRs: Untranslated regions
NSP: Non-structural proteins
ACE2: Angiotensin-converting enzyme 2

## References

[1] Chan JFW, Yuan S, Kok KH, To KKW, Chu H, Yang J (2020). A familial cluster of pneumonia associated with the 2019 novel coronavirus indicating person-to-person transmission: a study of a family cluster. Lancet.

[2] Li Q, Guan X, Wu P, Wang X, Zhou L, Tong Y (2020). Early transmission dynamics in Wuhan, China, of novel coronavirus-infected pneumonia. N Engl J Med.

[3] Lipsitch M, Swerdlow DL, Finelli L (2020). Defining the epidemiology of Covid-19—studies needed. N Engl J Med.

[4] Coronavirus Resource Center. https://coronavirus.jhu.edu/map.html. Accessed Mar 2.

[5] Cereda D, Tirani M, Rovida F, Demicheli V, Ajelli M, Poletti P, Trentini F, Guzzetta G, Marziano V, Barone A, Magoni M, Deandrea S, Diurno G, Lombardo M, Faccini M, Pan A, Bruno R, Pariani E, Grasselli G, Piatti A, Gramegna M, Baldanti F, Melegaro A, Merler S. The early phase of the COVID-19 outbreak in Lombardy, Italy. arXiv:2003.09320 [q-bioPE]. 2020.

[6] Lu R, Zhao X, Li J, Niu P, Yang B, Wu H (2020). Genomic characterisation and epidemiology of 2019 novel coronavirus: implications for virus origins and receptor binding. Lancet.

[7] Bar-On YM, Flamholz A, Phillips R, Milo R (2020). SARS-CoV-2 (COVID-19) by the numbers. Elife.

[8] https://www.gisaid.org G.

[9] Rambaut A, Holmes EC, O'Toole A, Hill V, McCrone JT, Ruis C (2020). A dynamic nomenclature proposal for SARS-CoV-2 lineages to assist genomic epidemiology. Nat Microbiol.

[10] Korber B, Fischer WM, Gnanakaran S, Yoon H, Theiler J, Abfalterer W (2020). Tracking changes in SARS-CoV-2 spike: evidence that D614G increases infectivity of the COVID-19 virus. Cell.

[11] Plante JA, Liu Y, Liu J, Xia H, Johnson BA, Lokugamage KG (2020). Spike mutation D614G alters SARS-CoV-2 fitness. Nature.

[12] Tegally H, Wilkinson E, Giovanetti M, Iranzadeh A, Fonseca V, Giandhari J (2020). Emergence and rapid spread of a new severe acute respiratory syndrome-related coronavirus 2 (SARS-CoV-2) lineage with multiple spike mutations in South Africa. medRxiv.

[13] Faria NR, Claro IM, Candido D, Moyses Franco LA, Andrade PS, Coletti TM, Silva CAM, Sales FC, Manuli ER, Aguiar RS, Gaburo N, Camilo CdC, Fraiji NA, Esashika Crispim MA, Carvalho MdPSS, Rambaut A, Loman N, Pybus OG, Sabino EC, on behalf of CADDE Genomic Network. Genomic characterisation of an emergent SARS-CoV-2 lineage in Manaus: 293 preliminary findings. 2021. https://virological.org/t/genomic-characterisation-of-an-emergent-294sars-cov-2-lineage-in-manaus-preliminary-findings/586.

[14] De Sabato L, Vaccari G, Knijn A, Ianiro G, Di Bartolo I, Morabito S (2021). SARS-CoV-2 RECoVERY: a multi-platform open-source bioinformatic pipeline for the automatic construction and analysis of SARS-CoV-2 genomes from NGS sequencing data. bioRxiv.

[15] Nguyen LT, Schmidt HA, von Haeseler A, Minh BQ (2015). IQ-TREE: a fast and effective stochastic algorithm for estimating maximum-likelihood phylogenies. Mol Biol Evol.

[16] Rakha A, Rasheed H, Batool Z, Akram J, Adnan A, Du J (2020). COVID-19 variants database: a repository for human SARS-CoV-2 polymorphism data. bioRxiv.

[17] Capobianchi MR, Rueca M, Messina F, Giombini E, Carletti F, Colavita F (2020). Molecular characterization of SARS-CoV-2 from the first case of COVID-19 in Italy. Clin Microbiol Infect.

[18] Lo Presti A, Rezza G, Stefanelli P (2020). Selective pressure on SARS-CoV-2 protein coding genes and glycosylation site prediction. Heliyon.

[19] Benvenuto D, Giovanetti M, Ciccozzi A, Spoto S, Angeletti S, Ciccozzi M (2020). The 2019-new coronavirus epidemic: evidence for virus evolution. J Med Virol.

[20] Saha P, Banerjee AK, Tripathi PP, Srivastava AK, Ray U (2020). A virus that has gone viral: amino acid mutation in S protein of Indian isolate of coronavirus COVID-19 might impact receptor binding, and thus, infectivity. Biosci Rep.

[21] Dawood AA (2020). Mutated COVID-19 may foretell a great risk for mankind in the future. New Microbes New Infect.

[22] Sheikh JA, Singh J, Singh H, Jamal S, Khubaib M, Kohli S (2020). Emerging genetic diversity among clinical isolates of SARS-CoV-2: lessons for today. Infect Genet Evol.

[23] van Dorp L, Acman M, Richard D, Shaw LP, Ford CE, Ormond L (2020). Emergence of genomic diversity and recurrent mutations in SARS-CoV-2. Infect Genet Evol.

[24] Mathavan S, Kumar S (2020). Evaluation of the effect of D614g, N501y and S477n mutation in Sars-Cov-2 through computational approach. Preprints.

